# Transylvania Medical School

**Published:** 1850-11

**Authors:** 


### THE WESTERN JOURNAL.

THIRD SERIES.—VOL. VI.—NO. V.

LOUISVILLE, NOVEMBER 1, 1850.

### TRANSYLVANIA MEDICAL SCHOOL.

This ancient and once renowned institution is certainly in a strange predicament. During the last winter, reports spread all over the country that its faculty were anxious to abandon it, and in the spring the professor, who was looked upon as the soul of the institution, did actually resign. The professors were understood to have reported to the board of trustees that the school was in a very languishing state—that its classes had run down to about fifty pay-pupils, and that it was useless to try to keep it up any longer in Lexington. In a word, it was universally regarded as in a dying condition. When the citizens of that place, a few months afterwards, were interrogated on the subject, they replied that the school was extinct. An old friend of the institution, and once a professor in it, in due time, announced its "dissolution," in his Medical Journal, at Cincinnati, concluding his somewhat pathetic obituary in the following words: "Thus, Transylvania, with all of its ancient renown, yielded slowly to the influence of circumstances beyond her control, until, finally, *she has fallen, to rise no more.*" We came, in turn, to notice the melancholy event, and after our poor fashion pronounced our eulogy upon the great defunct. And yet, after all, it turns out that the school claims to be alive. A writer in the October number of the *Transylvania Journal,* who signs himself "P.," asserts roundly that it is not dead, but that "the chairs are *all* filled with *able* teachers," he himself being one of the number!

Really, this appears a hard case. Is it possible that "P." is *Dr. Partridge,* revived, "the famous astrologer, almanac-maker and cobbler," whose history has been left on record by Dean Swift? The worthy dean professed, as is known, to give the last hours of the unfortunate astrologer, and reports his dying confession; in which he makes him say that he "repented from the bottom of his heart his impositions upon the people," adding, "I wish I may not have done more mischief by my physic than my astrology." The bell was tolled for him; the undertakers prepared for his funeral; his coffin was made, and his grave dug. The unhappy man came out, at last, and protested vehemently that he was not dead. but he seems never to have succeeded in convincing his friends of the fact. He says, touchingly, "I could not stir out of my doors for the space of three months after this, but presently one comes to me in the street: Mr. Partridge, that coffin you was last buried in, I have not yet been paid for. Doctor, cries another dog, how do you think people can live by making graves for nothing? next time you die you may even toll out the bell yourself for Ned. A third rogue tips me by the elbow, and wonders how I have the conscience to sneak abroad without paying my funeral expenses. Lord, says one, I durst have swore that was honest Dr. Partridge, my old friend; but, poor man, he is gone. I beg your pardon, says another, you look so like my old acquaintance that I used to consult on some private occasions; but, alack, he has gone the way of all flesh. Look—look—look, cries a third, after a competent space of staring at me, would not one think our neighbor, the almanac-maker, was crept out of his grave, to take the other peep at the stars in this world, and show how much he is improved in fortune-telling, by having taken a a journey to the other?" Nay," he continues mournfully, "the very reader of our parish, a good, sober, discreet person, has sent two or three times for me to come and be buried decently, or send him sufficient reason to the contrary; or, if I have been interred in any other parish, to produce my certificate as the act requires."

Now, we are not going to be so unkind to this Dr. Partridge as were the friends of the ancient doctor; we shall not insist on a certificate from him. We shall not even require him to stan bawling before his door, as did his great prototype—"alive! alive! ho! no counterfeit, but all alive!" Nor shall we attempt an argument to show, that, the soul and body having parted, the school is, according to the definition of philosophers, dead; and that what appears to be it is now but "an uninformed carcass, walking still about, and calling itself Transylvania." But we do insist that Dr. "P." had no more right to scold us for falling into the common mistake that his school was dead, than had the "carcass" of his great original "to beat the poor boy who happened to pass by it in the street, crying, a full and true account of Dr. Partridge's death."

In the denial of Dr. "P.," he complains further, that we did not report the size of his last class correctly. All we can say is, that we gave the number upon the authority of himself and his colleagues, and they, we should think, ought to know. They told the trustees of the school that the number of their "paying students" was some fifty odd, and if there was any mistake about it, the fault is none of ours. As to what the people of the theatre call "*dead heads*"—names added for effect—dragged in to impose upon the community—if "P." thinks worth while to count them, why then we do not; that is all. If he can make anything out of them, we honestly confess that we cannot.

But "P." says, his pupils, the few that he had, were very much pleased with his school. We shall, most assuredly, not be so discourteous as to deny it. No doubt, the students have been highly pleased with the school; only, for several years past, they have taken rather an odd way to testify their satisfaction; they have been somewhat careful not to return to it themselves, nor to send their pupils thitherwards. Owing to this queer fancy of its friends, its classes, of late, have been growing "small by degrees, and beautifully less;" until, really, every body had come to believe that it was on its last legs. Its worst enemy, if enemy it have, might now, we are sure, take up the words, in sincerity, "May its shadow never grow less!"

